# Linking Emotion Regulation Capacity and the Frequency of Daily Stressors in Old and Very Old Age

**DOI:** 10.1093/geroni/igab046.1279

**Published:** 2021-12-17

**Authors:** Martin Katzorreck, Denis Gerstorf, Anna Lücke, Hans-Werner Wahl, Oliver Schilling, Ute Kunzmann

**Affiliations:** 1 University of Leipzig, Leipzig, Sachsen, Germany; 2 Humboldt University of Berlin, Humboldt University of Berlin, Brandenburg, Germany; 3 Heidelberg University, Heidelberg, Baden-Wurttemberg, Germany; 4 University of Heidelberg, Heidelberg, Baden-Wurttemberg, Germany; 5 Leipzig University, Leipzig, Sachsen, Germany

## Abstract

Lifespan theories and lab-based research both suggest that the ability to downregulate negative emotions is often well preserved into old age, but becomes increasingly fragile in very old age. However, little is known about factors that may alleviate such age differences. Here, we ask whether exposure to daily stressors helps very old adults to maintain effective emotion regulation skills. We used data from 130 young-old (65-69 years, 48% women) and 59 very-old adults (83-89 years, 58% women) who watched negative emotion evoking film clips in the lab under emotion regulation instructions and also reported stress situations they experienced in everyday life (42 occasions across seven days). Initial results indicate that very-old adults were indeed less successful in regulating sadness than young-old adults, but those very-old adults who reported many daily stressful situations were as capable of emotion regulation as young-old adults. We discuss possible factors contributing to our age-differential findings.

